# Emotion Dysregulation and Parent Emotion Socialization in Mothers with Borderline Pathology

**DOI:** 10.21203/rs.3.rs-6355485/v1

**Published:** 2025-05-09

**Authors:** Ashley Lubben, Tess Gecha, Kiana Cano, Carla Sharp

**Affiliations:** University of Houston; University of Houston; Sam Houston State University; University of Houston

**Keywords:** borderline personality disorder, subclinical borderline personality disorder, parent emotion socialization, emotion dysregulation, parenting with borderline personality disorder

## Abstract

**Background:**

Mothers with borderline personality disorder face unique challenges in parenting, as borderline symptoms have been shown to negatively affect parent-child relationships. These challenges can lead to non-supportive reactions to children’s negative emotions, a form of parent emotion socialization (PES) that has been linked to negative outcomes in children. Given the inherent emotional arousal evoked by parenting, emotion dysregulation likely influences the type of PES parents utilize. However, no studies have specifically examined how emotion dysregulation affects PES in mothers with borderline pathology. Against this background, this study aims to (1) investigate the link between maternal emotion dysregulation and PES strategies and (2) assess if emotion dysregulation mediates the relationship between borderline pathology and PES.

**Methods:**

The study sample was comprised of 148 mothers (*Mage* = 34.92). Of these mothers, 53 had significant borderline features. Emotion regulation was evaluated using the Difficulties in Emotion Regulation Scale – Short Form and PES was assessed using the Coping with Children’s Negative Emotion Scale. Significant borderline features were determined using the Personality Assessment Inventory Borderline Scale. As a part of aim 1, bivariate correlations were conducted to examine relationships between emotion dysregulation and two PES strategies: supportive and non-supportive. The moderating role of emotion dysregulation on the relationships between borderline features and supportive and non-supportive PES was assessed using two moderation models.

**Results:**

Results from the first aim revealed a small, negative correlation between emotion dysregulation and supportive PES and a medium, positive correlation between emotion dysregulation and non-supportive PES. Emotion dysregulation was found to be a significant moderator of the relationship between borderline pathology and non-supportive PES.

**Conclusions:**

The current study significantly contributes to the literature by further elucidating the relationship between maternal borderline pathology and PES and its underlying mechanisms.

## Background

Borderline personality disorder (BPD) is a psychiatric disorder involving high instability in relationships, mood lability, and impulsive behaviors [1]. Emotion dysregulation, a central feature of BPD, may lead to disproportionate emotional reactions—a behavior that extends to parenting contexts. Research increasingly indicates that mothers with BPD struggle with parent emotion socialization (PES) [2, 3], which refers to how parents shape their children’s emotional understanding, expression, and regulation through supportive or non-supportive behaviors [4]. Effective PES relies on the accurate identification of a child’s negative emotions, allowing for appropriate and supportive responses. However, mothers with BPD often struggle to distinguish and identify their children’s emotions [5, 6]. This impairment, coupled with maladaptive parenting behaviors associated with this population [6–8], increases the likelihood of negative responses, potentially hindering their child’s development.

Emotion regulation can broadly be defined as an individual’s capacity to manage their emotions and behaviors in situationally appropriate ways, particularly in distressing circumstances [10, 11]. Emotion regulation is imperative for adaptive parenting, especially in high arousal situations such as when their child is in distress [12]. If a mother is unable to regulate these intense emotions, she is more likely to engage in non-supportive parenting behaviors [13–15]. As emotion dysregulation and BPD are strongly associated [11, 16], mothers with BPD may be especially likely to display these behaviors. Previous research has demonstrated significant correlations between BPD, non-adaptive parenting, and maternal emotion regulation [2, 17–20]. However, there remain few studies investigating the interplay between these three constructs.

While studies like Kiel’s study conducted in 2017 [2] have explored how mothers with BPD regulate emotions in response to their infants, no current study focuses on the connection between emotional regulation in BPD mothers and young children’s negative emotional expression. Against this background, the current study used a sample of mothers with and without borderline pathology to examine whether emotion dysregulation is correlated with supportive and/or non-supportive parent emotion socialization (PES). Additionally, moderation analyses were performed to assess the moderating role of emotion dysregulation on the relationship between borderline pathology and PES strategies.

## Methods

### Participants

The participants of the current study consisted of two groups of mothers: mothers exhibiting borderline pathology and healthy controls. This dataset was obtained from a larger study examining the viability of the Mediational Intervention for Sensitizing Caregivers for mothers with borderline personality disorder (BPD) [21]. The Institutional Review Board (IRB) at University of Houston reviewed and approved study procedures. Participants for this study were recruited through postings online in national BPD support groups. Eligibility criteria included being English-speaking, over the age of 18, and the mother of at least one child under the age of 18.

The final sample included 148 participants. Of those, 53 had scores indicating significant borderline features according to the Personality Inventory Assessment - borderline scale (PAI-BOR) [22]. These mothers represented the BPD sample. Mothers ranged in age from 21 to 53 years old (*M* = 34.92, *SD* = 6.27) and their children of interest ranged from 2 to 12 years old (*M* = 6.42, *SD* = 3.17). Mothers primarily identified as Caucasian/White (83.1%, *n* = 123), married (62.2%, *n* = 20) with an annual income of greater than $60,000 (58.1%, *n* = 86). Most participants (82.4%, *n* = 122) reported previously receiving mental health treatment and about half (50.7%, *n* = 75) were currently receiving treatment. Full demographic information is reported in Table 1.

Participants first received a cover letter outlining the aims of the study, confidentiality measures, potential risks and benefits, compensation details, withdrawal options, and contact information. Participants then indicated willingness to participate, and eligibility was determined by screening questions. Upon consenting and confirming their eligibility, they were provided with a set of Qualtrics surveys and instructed to respond to all questions with a single child in mind to maintain consistency across all survey measures. Participants also indicated whether they would like to be entered into a raffle for a chance to win one of fifty $20 Amazon gift cards after completing the surveys.

## Measures

### Parent Emotion Socialization

#### Child’s Coping With Negative Emotions Scale (CCNES) [23]

The CCNES is a self-report scale that aims to assess how parents respond to their young children when they communicate distressed or unpleasant feelings, which represents a form of PES. The measure presents 12 hypothetical, emotionally evocative scenarios that children commonly undergo. For each scenario, participants use a 7-point Likert scale to rate their likelihood of engaging in six different types of responses to their child’s behavior. Each type of response represents a different subscale, including problem-focused reactions (PFR), emotion-focused reactions (EFR), expressive encouragement (EE), minimization reactions (MR), punitive reactions (PR), and distress reactions (DR). These subscales can be synthesized into aggregate means representing supportive PES, consisting of PFR, EFR, and EE, and non-supportive PES, consisting of MR, PR, and DR. The CCNES has been shown to be a reliable measure, exhibiting consistent tendencies over time and displaying connections with related measures of concurrent validity [24]. In the current sample, Cronbach’s alpha indicated good internal consistency for non-supportive subscales (a =.82) and excellent internal consistency for supportive subscales (a =.91).

### Emotion Dysregulation

#### Difficulties in Emotion Regulation Scale- short form (DERS-SF) [10]

DERS-SF is an 18-item self-report measure consisting of statements on a 5-point Likert scale, ranging from almost never to almost always, designed to assess emotion dysregulation in adult populations. Its six subscales, which include nonacceptance of emotional responses, difficulty engaging in goal-directed behavior, impulse control difficulties, lack of emotional awareness, limited access to emotion regulation strategies, and lack of emotional clarity, can be combined to yield a total score of difficulties with emotion regulation. DERS-SF is a sound measure of emotion dysregulation displaying good reliability, convergent validity, and invariance across genders [25, 26]. In the current sample, Cronbach’s alpha showed excellent internal consistency for the DERS-SF measure (a =.91).

### Borderline Pathology

#### Personality Assessment Inventory – borderline scale (PAI-BOR) [22]

PAI-BOR is a subsection of the larger Personality Assessment Inventory (PAI), a comprehensive self-report measure of various domains of psychopathology, that measures borderline personality pathology. PAI-BOR is one of 11 clinical scales included in the PAI consisting of 24 items, all of which are answered on a 4-point Likert scale from “very false” to “very true”. Its four subscales, which include affective instability, identity problems, negative relationships, and self-harm, can be combined to yield a total continuous score of BPD symptomology. Furthermore, the manual of the PAI-BOR identifies a raw score of 38 or more as an indicator of significant borderline features. Previous studies support the criterion, concurrent, and external validity of the PAI measure for borderline symptomology [27, 28]. In the current sample, PAI-BOR demonstrated good internal consistency (a =.83).

### Data Analytic Strategy

All data analyses for this study were conducted using the IBM SPSS software version 25.0 [29]. First, descriptive statistics and bivariate relations among main study variables were conducted to identify potential covariates, including mother age, child age, and child gender.

To test aim 1, we conducted bivariate correlations to examine the relations between emotion dysregulation, as measured by the DERS-SF, and parent emotion socialization (PES) strategies, quantified by the CCNES measure. To test aim 2, we created a binary variable assessing whether mothers had levels of borderline pathology meeting the subclinical threshold of the PAI-BOR measure, while the DERS-SF measure remained continuous. This approach was chosen due to significant overlap between the PAI-BOR and DERS-SF measures (see Table 2). SPSS PROCESS macro [30] was used to examine whether the total emotion dysregulation score moderates the relationship between BPD symptomatology and the aggregate means of supportive and non-supportive PES. Additionally, the regression analysis controlled for any covariates found in initial analyses.

## Results

### Aim 1. Correlations between Emotion Dysregulation and Parent Emotion Socialization

The first aim of the study sought to assess the relationship between emotion dysregulation and parent emotion socialization (PES) within a combined sample of mothers with borderline features and healthy control mothers. Descriptive statistics and Pearson’s correlations are displayed in Tables 1 and 2, respectively.

Several covariates emerged as statistically significant. As shown in Table 2, mother’s age showed a significant negative correlation with DERS-SF total score (*r* = −.40, p <.001) and PAI-BOR total score (*r* = −.36, *p* <.001), while child’s age revealed a significant positive relationship with non-supportive PES (*r* =.30, *p* <.001). Regarding relations between demographic variables and PES strategies, Pearson’s correlations revealed no significant correlations between child age or gender and specific PES strategies (See Table 2).

Significant correlations were found between several variables of interest. As displayed in Table 2, DERS-SF total score showed a significant negative correlation of small effect with supportive PES strategies, whereas there was a significant positive correlation of medium effect with non-supportive PES strategies.

### Aim 2. The Moderating Effect of Emotion Dysregulation on BPD Symptomology on Parent Emotion Socialization

**Figure 1:**
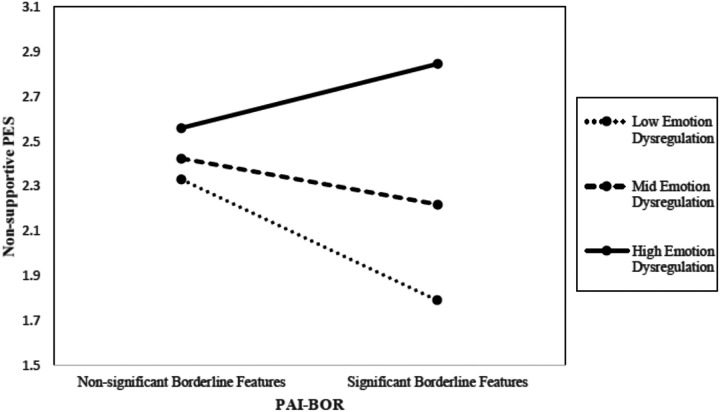
The moderating effect of DERS-SF on the relationship between PAI-BOR and non-supportive PES

The second aim of the study was to examine how emotional dysregulation moderates the relationship between presence of BPD features and specific PES strategies. Table 2 displays the bivariate correlations among study variables, indicating that meeting the subclinical threshold on PAI-BOR was significantly correlated with non-supportive PES strategies, but not with supportive ones. Given that both mother’s age and child’s age were significantly correlated with the independent and dependent variables of interest, respectively, we included the variables as covariates in both moderation analyses. While total score for emotion dysregulation was not found to moderate the relationship between borderline pathology and supportive PES, it did emerge as a significant moderator for the relationship between borderline pathology and non-supportive PES (See Fig. 1). More specifically, the interaction between borderline features and DERS-SF total score on supportive PES produced nonsignificant results (*b* = −.01, SE =.01, *p* =.54). However, there was a significant interaction of small effect size between PAI-BOR and DERS-SF for the outcome of non-supportive PES (*b* =.03, *SE* =.01, *T* = 2.68, *p* <.01). Results of these analyses are reported in Table 3.

## Discussion

The present study aimed to further investigate the relationship between borderline pathology, emotion regulation, and parent emotion socialization (PES) strategies utilized by mothers with borderline pathology. The findings of this study contribute to current literature suggesting that mothers with borderline personality disorder (BPD) struggle to parent effectively and adaptively, particularly in domains of parenting that require increased emotion regulation and understanding [7, 31]. Previous research has demonstrated how mothers with BPD have a higher likelihood of using non-supportive parenting behaviors [7, 8, 32]. One such study, Kiel et al. (2017), found that in mother-infant dyads where the mother was diagnosed with BPD, the correlation between BPD features and non-supportive PES was mediated by emotion dysregulation [2]. However, no previous study has investigated the relationships among these constructs in mothers with children beyond infancy.

With respect to aim 1, we hypothesized that greater difficulties in emotion regulation as measured by the DERS-SF would be positively associated with non-supportive PES strategies and negatively associated with supportive PES strategies. In line with this hypothesis, DERS-SF scores were found to have a significant positive correlation with non-supportive PES and a significant negative correlation with supportive PES. These findings corroborate existing research demonstrating how parental difficulties with emotion regulation are correlated with an increased use of nonadaptive or non-supportive parenting behaviors [2, 12, 17, 33]. Further, previous studies investigating these variables affirm a significant negative correlation between maternal difficulties in emotion regulation and positive PES behaviors [33].

Parenting places significant emotional demands on mothers and can thus be affectively challenging and destabilizing, especially for mothers with borderline pathology who tend to struggle with emotion regulation. Parents need to successfully regulate their own emotions to engage in adaptive parenting behaviors. The results of this study demonstrate how increased difficulty with emotion regulation is related to both increased non-supportive PES and decreased supportive PES. However, underlying constructs that might help explain these relationships are still largely unknown. One construct that might subserve the relationship between emotion regulation and PES is mentalization. Mentalization is the ability to conceptualize the mental states of oneself and others [34]. A closely related construct is theory of mind, also referred to as cognitive empathy, which uniquely focuses on understanding the mental states of others [35, 36]. Decety’s neurodevelopmental model of empathy posits that emotional arousal informs our understanding and representation of others’ mental states [37, 38]. Therefore, emotion dysregulation leads to misinterpretation of others’ mental states. This theory is supported by previous research indicating that mothers with higher levels of emotional suppression and dysregulation are associated with impaired mentalization [35, 39].

Our second aim was to investigate the moderating effect of emotion regulation difficulties on the relationship between borderline pathology and PES strategies. We hypothesized that emotion dysregulation would moderate the relationship between borderline pathology and PES such that mothers with subclinical borderline pathology and more difficulties in emotion regulation would engage in less supportive and more non-supportive PES. The former of these hypotheses produced null findings—emotion dysregulation did not moderate the BPD-supportive PES relationship. However, difficulties in emotion regulation did moderate the relationship between borderline symptomatology and non-supportive PES. This discrepancy in results may be indicative of disorganized parenting behaviors from mothers with BPD. Whilst both healthy controls and mothers with BPD display supportive PES behaviors, mothers with BPD also display non-supportive PES to a significant degree. Current literature has shown that mothers with BPD oscillate between extreme behaviors on various continuums—between overly lax and overly controlling and between warm and hostile [7, 9]. Our results indicate that PES represents another domain of fluctuating parenting behavior for these mothers, one that is moderated by the mothers’ individual differences in emotion regulation.

Additionally, other features of BPD like impulsiveness and impaired mentalization may contribute to the relationship between borderline pathology and non-supportive PES. According to Fonagy and Bateman’s mentalization model of BPD (2008), heightened emotional sensitivity in BPD causes hyperarousal during stress, particularly in attachment contexts, making it more difficult for mothers to identify and react effectively to their child’s negative emotional expression. Sharp’s (2014) hypermentalizing theory posits that individuals with borderline pathology over-attribute mental states to others, leading to socio-cognitive difficulties and interpretive errors during emotionally intense interactions [40]. This might lead to mothers misinterpreting their child’s feelings, resulting in inappropriate, non-supportive responses that can perpetuate frustration and misidentification. At the same time, a core feature of BPD is impulsivity [1], which is exacerbated by emotion dysregulation [41, 42]. Emotion dysregulation has been found to subserve the relationship between BPD and various domains of impulsivity [41], and both factors may contribute to interpersonal problems [43]. In this way, emotion dysregulation’s association with heightened impulsivity as well as impaired mentalization may explain why mothers with borderline pathology might employ more non-supportive PES strategies when reacting to a distressing situation like responding to their children’s negative emotions.

### Clinical Implications

The results of this study have significant implications for clinical practice. Abundant research has shown that supportive parent emotion socialization (PES) strategies foster healthier emotional development in children [12]. Findings from our study suggest that treatment focused on the improvement of emotion regulation abilities in mothers may aid maternal emotion regulation whilst leading to adaptive PES behaviors. This in turn will likely result in better emotional health and understanding for both parent and child. Interventions such as dialectical behavior therapy (DBT) [44] or mentalization-based therapies (MBT) [45] may be especially helpful for mothers with borderline pathology or mothers with heightened difficulties regulating their emotions. DBT, which focuses on mindfulness and emotion regulation, has been shown to effectively reduce borderline symptoms, particularly emotion dysregulation [44, 46]. MBT aims to stabilize emotional arousal and improve understanding of mental states, enhancing outcomes for patients with borderline pathology, especially when combined with DBT [45, 47].

### Limitations and Future Directions

Our current study contributes to the field by providing new and relevant perspectives on how emotion regulation difficulties in mothers with borderline pathology influence parent emotion socialization strategies. However, the present study is not without its limitations. Firstly, our reliance on self-report measures introduces potential bias, which may be compounded by impaired mentalizing capacity associated with BPD. Additionally, our sample was predominantly non-Hispanic white, which limits the generalizability of our findings to other populations. Finally, our cross-sectional design limits our ability to assess causal relationships. Future research should investigate the relationship between these variables using a more diverse sample and a longitudinal design. This approach would allow for a more nuanced and generalizable analysis that accounts for situational stressors influencing mothers’ mental states over time, as well as treatment effects that may alter their emotion regulation and parenting behaviors.

## Conclusion

The current study significantly contributes to the literature by further elucidating the relationship between maternal borderline pathology and parent emotion socialization (PES). This is the second study to investigate the relationship between emotion dysregulation, maternal borderline pathology, and PES [2] and the first to do so with a sample of mothers of children. Our findings contribute to the current understanding of how emotion dysregulation and borderline pathology influence maternal behaviors that shape the emotional development of their children. Furthermore, these findings underscore the importance of addressing emotion dysregulation in parenting interventions—especially for mothers with borderline pathology—to promote healthier emotional outcomes for both mother and child. Taken together, results suggest that increasing difficulties with emotion regulation is associated with increased use of non-supportive PES strategies and decreased use of supportive PES behaviors. Results also indicate that difficulties in emotion regulation moderate the relationship between borderline pathology and non-supportive, but not supportive, PES. Future research should expand on these findings by incorporating additional mechanisms affecting PES, such as impulsivity and mentalization. Efforts should also include a more diverse sample and employ a longitudinal design to better understand how these factors evolve over time and impact PES behaviors in mothers with borderline pathology.

## Figures and Tables

**Table 2.** Pearson correlations among variables of interest

	1.	2.	3.	4.	5.	6.	7.
**Difficulties in emotion regulation**							
1. DERS-SF Total Score							
**Parent emotion socialization**							
2. Supportive	−.20*						
3. Non-supportive	.39**	−.27**					
**Personality Assessment Inventory- Borderline Scale**							
4. PAI-BOR Total Score	.82**	−.08	.27**				
**Demographics**							
5. Mother age	−.40**	−.06	.01	−.36**			
6. Child age	.12	−.11	.30**	.13	.40**		
7. BPD diagnosis	.66**	−.06	.13	.72**	−.34**	−.02	

Note.

* *p* <.05,

** *p*<.01

**Table 3.** Moderation analyses

Moderating effect of DERS-SF on the relationship between PAI-BOR and supportive PES				
	b	SE β	t	p

**Constant**	6.92	.50	13.76	< .0001
**Presence of BPD features**	.71	.60	1.19	.24
**DERS-SF total**	−.02	.01	−2.41	.02
**PAI-BOR x DERS-SF**	−.01	.01	−.61	.54
**Child age**	−.01	.02	−.39	.70
**Mother age**	−.01	.01	−1.29	−.04
Moderating effect of DERS-SF on the relationship between PAI-BOR and non-supportive PES				
	b	SE β	t	p
**Constant**	1.48	.43	3.39	< .001
**Presence of BPD features**	−1.32	.52	−2.54	.01
**DERS-SF total**	.01	.01	1.16	.25
**PAI-BOR x DERS-SF**	.026	.01	2.68	< .01
**Child age**	.05	.02	2.82	.01
**Mother age**	.01	.01	.92	.36

## Data Availability

Requests for access to data can be sent to Dr. Carla Sharp (csharp2@central.uh.edu).

## Abbreviations

BPD: Borderline personality disorder
CCNES: Child’s Coping with Negative Emotions Scale
DERS-SF: Difficulties in Emotion Regulation Scale- Short Form
PES: Parent emotion socialization
PAI-BOR: Personality Assessment Inventory- Borderline Scale
